# Burden and Health System Challenges in MASLD Across East and Southeast Asia: A Narrative Review

**DOI:** 10.1002/hsr2.72304

**Published:** 2026-04-15

**Authors:** Jonathan Jaime G. Guerrero, Paolo C. Encarnacion, Wan‐Yu Wang, Shu‐Fen Liao, Ching‐Wen Chang

**Affiliations:** ^1^ College of Medicine University of the Philippines Manila Philippines; ^2^ College of Public Health University of the Philippines Manila Philippines; ^3^ Graduate Institute of Metabolism and Obesity Sciences Taipei Medical University Taipei Taiwan; ^4^ Department of Industrial Engineering and Management Yuan Ze University Taoyuan Taiwan; ^5^ Division of General Surgery, Department of Surgery, Shuang Ho Hospital Taipei Medical University Taipei Taiwan; ^6^ School of Public Health, College of Public Health Taipei Medical University Taipei Taiwan; ^7^ Department of Medical Research, Wan Fang Hospital Taipei Medical University Taipei Taiwan; ^8^ TMU Research Center for Digestive Medicine Taipei Medical University Taipei Taiwan; ^9^ Taipei Cancer Center Taipei Medical University Taipei Taiwan

**Keywords:** East Asia, epidemiology, MASLD, metabolic dysfunction, public health, Southeast Asia

## Abstract

**Background and Aims:**

Metabolic dysfunction–associated steatotic liver disease (MASLD) is an escalating public health concern in East and Southeast Asia, driven by rapid urbanization, dietary transitions, and rising rates of obesity, diabetes, and hypertension. Unique metabolic and phenotypic characteristics among Asian populations, coupled with resource constraints, contribute to underdiagnosis and delayed management. This narrative review aims to examine the burden, risk factors, population‐specific challenges, and public health gaps related to MASLD across East and Southeast Asian countries.

**Methods:**

A comprehensive literature search was conducted using PubMed, ScienceDirect, and Google Scholar for studies published from 2010 to 2024. Search terms included “MASLD,” “MAFLD,” “NAFLD,” “fatty liver disease,” “East Asia,” “Southeast Asia,” “risk factors,” and “public health.” Eligible studies included original research, cohort studies, reviews, and meta‐analyses focused on MASLD or NAFLD in the specified regions. Data were synthesized thematically, emphasizing prevalence patterns, lifestyle determinants, vulnerable populations, and health‐system challenges.

**Results:**

MASLD prevalence varies widely across East and Southeast Asia, with higher rates observed in urban and high‐income settings. Beyond metabolic drivers, environmental factors, dietary shifts, and socioeconomic disparities strongly influence disease patterns. Distinct subpopulations—including children, older adults, individuals with lean MASLD, and those with chronic comorbidities—experience unique vulnerabilities, often compounded by limited region‐specific data. Major health‐system gaps include inadequate integration of MASLD into primary care, variability in diagnostic access, insufficient public health surveillance, and low provider awareness.

**Conclusion:**

MASLD represents a growing yet underrecognized public health issue in East and Southeast Asia. Addressing diagnostic gaps, enhancing primary care capacity, and integrating MASLD within existing metabolic disease programs are essential for improving early detection and long‐term outcomes. Region‐specific strategies and strengthened health systems are critical to mitigating the future burden of MASLD across diverse Asian populations.

## Summary

**What is already known on this topic**

- MASLD is increasing in prevalence in East and Southeast Asia due to urbanization, dietary shifts, and rising metabolic diseases. However, it remains underdiagnosed and undertreated, especially in low‐resource settings, with limited research on its public health impact.

**What this study adds**

- This study highlights significant regional disparities in MASLD prevalence, healthcare access, and disease burden, particularly among vulnerable groups and the critical gaps, including the lack of integration into primary care, insufficient epidemiological data, and the absence of standardized screening protocols tailored to the region.

**How this study might affect research, practice or policy**

- This study underscores the urgent need for region and nation‐specific MASLD screening programs, integration into primary healthcare, and targeted public health interventions, and developing sustainable strategies to mitigate the long‐term socioeconomic and health burdens of MASLD in East and Southeast Asia.

## 1 Introduction

Metabolic dysfunction‐associated steatotic liver disease (MASLD), evolving from previous nomenclatures to better represent the paradigm shift in understanding and addressing fatty liver conditions [1, 2, 3], remains a significant global public health concern. As a multifaceted disease, MASLD encompasses a complex interplay of metabolic, genetic, environmental and lifestyle factors. Its pathogenesis is closely tied to metabolic dysfunction, including obesity, insulin resistance, type 2 diabetes, and dyslipidemia [4, 5, 6]. Beyond its hepatic impact, MASLD is associated with an increased risk of cardiovascular disease [7, 8] and chronic kidney disease [9], and risk for decreased bone mineral density [10], among other systemic complications. These associations underscore MASLD's significance as more than just a liver‐specific condition, perhaps effectively framing it as a systemic disorder with widespread implications for patient health and healthcare systems.

The 2023 consensus guidelines officially defined MASLD as a liver disease characterized by hepatic steatosis in the presence of metabolic dysfunction. The diagnosis of MASLD also requires the presence of at least one of the five cardiometabolic risk factors in the context of hepatic steatosis with no other discernible cause of such steatosis [2, 3, 11]. If there are other additional drivers of steatosis, this can be considered as a combination etiology [12], as in the case of hepatitis infection and MASLD occurring together.

While we continue to discover more about the disease, the pathogenesis of MASLD has arguably been elucidated well in literature [13, 14, 15]. Clinically, we understand it as a progressive condition characterized by the excessive accumulation of fat in liver cells, often without significant alcohol consumption. Over time, this benign state evolves into more severe forms such as steatohepatitis, fibrosis, cirrhosis and even hepatocellular carcinoma [16]. From a molecular perspective, lipid accumulation leads to cellular stress and generation of reactive oxygen species, which triggers mitochondrial dysfunction and endoplasmic reticulum stress [15, 17, 18, 19]. Recent studies have also highlighted the role of genetic and epigenetic factors, such as variants in PNPLA3, TM6SF2 and HSD17B13 genes, which influence susceptibility to MASLD and its progression [20, 21, 22, 23].

Much is known of MASLD at an individual level in terms of clinical manifestations and pathogenesis. However, it is important to expand this understanding to a broader public health lens that addresses the systemic, societal and environmental factors contributing to its rising prevalence. In this narrative review, we zoom out to the population level, focusing on the broader public health impact of MASLD, particularly within the context of the East and Southeast Asian populations. These populations are of particular interest due to their unique epidemiological and genetic profiles, as well as the diverse and rapid lifestyle and dietary transitions within the region. We focus on its epidemiology, contributing social and environmental factors and gaps in public health responses.

## 2 Methodology

This narrative review employed a structured approach to identify, select, and synthesize relevant literature on metabolic dysfunction–associated steatotic liver disease (MASLD) in East and Southeast Asia. A comprehensive search of peer‐reviewed articles was conducted using three major electronic databases—PubMed, ScienceDirect, and Google Scholar—from January 2010 to December 2024, ensuring inclusion of contemporary studies aligned with evolving nomenclature and diagnostic frameworks. Search terms combined controlled vocabulary and free‐text keywords related to MASLD and regional epidemiology. Core search strings included: “MASLD,” “MAFLD,” “NAFLD,” “hepatic steatosis,” “fatty liver disease,” “cardiometabolic risk,” “East Asia,” “Southeast Asia,” “prevalence,” “genetics,” “risk factors,” “diet,” and “public health.” Boolean operators and truncation (e.g., *MASLD* AND *Asia*, *fatty liver* AND *genetic variants* AND *Southeast Asia*) were applied to broaden or refine results.

Studies were included if they met the following criteria: (1) published in English; (2) original research, meta‐analyses, narrative or systematic reviews, cohort studies, or population‐based reports; (3) focused on MASLD/NAFLD epidemiology, genetics, metabolic risk factors, population‐specific characteristics, or health‐system challenges in East or Southeast Asian countries; and (4) provided human data. Exclusion criteria were: (1) animal‐only studies, (2) case reports with insufficient generalizability, (3) studies outside the geographic scope, and (4) papers lacking relevance to MASLD or its cardiometabolic and public‐health dimensions.

The selection process followed an iterative review of titles, abstracts, and full texts. Reference lists of key articles were hand‐searched to identify additional eligible studies. Data were extracted and synthesized thematically, focusing on epidemiology, genetic and lifestyle determinants, associations with cardiovascular and metabolic diseases, vulnerable subpopulations, and gaps in public health systems across the region. As this work is a narrative review, quantitative meta‐analysis was not performed; instead, emphasis was placed on integrating heterogeneous evidence to provide a region‐specific, contextual understanding of MASLD.

### 2.1 Country Prevalence of MASLD and Associated Diseases

**Figure 1:**
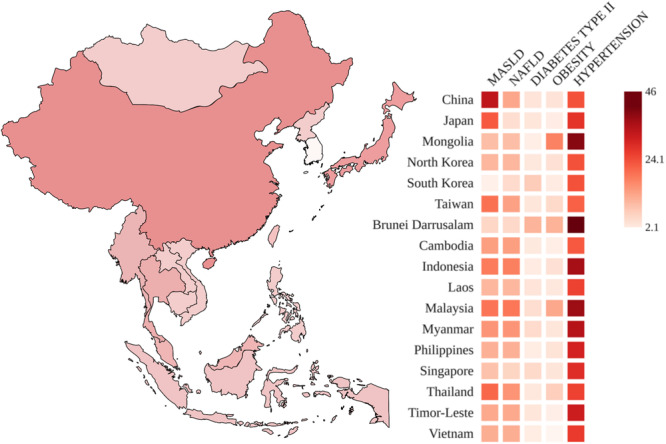
Prevalence of MASLD (geographic heat map) and associated diseases across countries of East and Southeast Asia. Darker shading indicates higher reported burden within each mapped indicator. Detailed country‐specific values, data sources, and indicator definitions are provided in Supporting Table 1.

The prevalence of MASLD in Southeast Asia (Figure 1) is estimated to be at 33.1% while 29.7% in East Asia, roughly reflecting the global prevalence of the disease in 2019 [24]. In China, prevalence of the disease is at 36.91% [25], with urban areas possibly experiencing the highest rates due to sedentary lifestyles and high‐calorie diets. In high‐income countries such as Japan, prevalence ranges from 26% [26] to around 28% [27]. Southeast Asian countries have limited data on the prevalence of MASLD per se, but non‐alcoholic fatty liver disease (NAFLD) data shows that Indonesia, Malaysia, and Thailand have the highest prevalence among their SEA neighbors [28]. Singapore and Brunei report a prevalence similar to other developed East Asian countries such as South Korea and Japan [28]. Prevalence values are shown in Supporting Table 1.

Gender differences are notable in some countries, with MASLD being more prevalent in males than in females, as in the case of Japan with 30.3% of MASLD patients are male compared to 16.1% female [29]. Age is another critical factor, with younger adults in urbanized areas of Southeast Asia such as Singapore, Malaysia and the Philippines being affected due to early‐onset obesity and lifestyle related metabolic disorders [30].

Of particular concern is the disease burden being disproportionately high among certain subpopulations. While we know that patients with diabetes, obesity, hypertension and cardiometabolic diseases have increased predisposition to MASLD, a few studies have looked into other chronic comorbidities such as HIV infection, cancer patients undergoing chemotherapy, and chronic kidney disease undergoing dialysis. These chronic diseases are prevalent in many parts of East and Southeast Asia which can complicate the management of MASLD in the long run. MASLD is highly prevalent among people living with HIV in Thailand, with about half with significant fibrosis [31]. Sarcopenia, a well‐documented effect of HIV infection, cancer and their treatments, is found to be associated with MASLD progression and poor prognosis among Korean, Chinese, and Japanese populations [32].

### 2.2 Risk Factors in the Asian Population

A confluence of genetic, lifestyle, and environmental factors unique to the region influence the progression of MASLD in Asian populations. In East and Southeast Asia, genetic predispositions play a significant role. At least 4 genome‐wide association studies studies have been conducted in the Asian population that reported PNPLA3, SAMM50, PARVB and GATAD2A genes that are significantly associated with NAFLD [33], which essentially predisposes the same to MASLD. In particular, PNPLA3 have strong associations among Japanese, Taiwanese, Korean and Chinese populations [34, 35, 36, 37, 38, 39, 40]. Among Filipinos, PNPLA3 genotypes and risk of developing NAFLD were found to be not significantly associated [41]. Among Thai populations, PNPLA3 and SIRT5 polymorphisms were independently and additively associated with fibrosis, while HSD17B13 polymorphism with increased steatosis [42]. Among Malaysians, single nucleotide polymorphism in the gene GCKR is associated with NAFLD, NASH, and fibrosis [43] while the same SNP is reported to have no association among Chinese population of Singapore [44], highlighting ethnic variability within Southeast Asia.

Dietary patterns in these regions, such as the high consumption of fiber‐rich foods and fermented foods have profound impact on gut microbial diversity. Bacterial genera, along with other factors, was independently associated with advanced fibrosis in Thai patients with MASLD [45]. Similar findings of gut dysbiosis in Indonesian patients significantly predisposes them to MASLD [46]. On the other hand, a dairy‐rich diet pattern among Koreans was significantly associated with lower risk of MASLD, while a carnivore, plant‐based, or starch‐rich diet pattern showed no significant association with MASLD incidence [47]. Among Japanese adults, eating noddles/rice bowl and evening meals often positively associated with non‐obese MASLD [48].

Lifestyle factors are equally critical, a fact not unique to the Asian population. The rapid urbanization of countries like China, Vietnam, and Indonesia has led to sedentary lifestyles and increased consumption of high‐calorie, low‐nutrient diets [49]. Smoking and alcohol consumption, including beer, are additional modifiable risk factors. Furthermore, variability of physical activities is reported across East and Southeast Asia — 37.0% in Singapore with high levels of sedentary behavior [50], 23.5% among Malaysian adults [51], to as high as 75.8% among Thai during the COVID‐19 lockdown [52], which can predispose patients to MASLD in the long run.

### 2.3 Complications and Association With Diseases

MASLD is associated with significant complications and mortality, primarily due to its progression to advanced liver diseases and its link to metabolic comorbidities. MASLD can progress to cirrhosis and hepatocellular carcinoma, which potentially increases the burden of HCC in countries such as Mongolia where highest incidence of HCC is recorded worldwide [53]. There is strong evidence for a causal association of MASLD and HCC among East Asians [54]. Likewise, it is associated with a number of extrahepatic cancers such as gastric, colorectal, and thyroid cancers, to name a few [55]. In numerous studies, NAFLD was independently associated with increased risk of colorectal adenomas, neoplasms or polyps among populations in China, Taiwan, and South Korea [56, 57, 58, 59]. MASLD also predisposes patients to complications and early mortality among groups with specific comorbidities. Among Japanese with inflammatory bowel disease (IBD), MASLD increases risk of cardiometabolic factors and mortality due to hepatic inflammation and fibrosis [60, 61]. The specificity of these complications and their unique impact on the Asian population requires further investigation.

MASLD has also been shown to exert an independent and clinically meaningful association with increased cardiovascular disease (CVD) risk [62, 63]. MASLD contributes to cardiometabolic stress beyond traditional risk factors such as hypertension, dyslipidemia, and diabetes, likely through systemic inflammation, insulin resistance, endothelial dysfunction, and pro‐atherogenic lipid remodeling. In Asians, the association is stronger due to unique metabolic phenotypes—such as increased visceral adiposity at lower BMI, higher susceptibility to insulin resistance, and genetic predispositions like PNPLA3 and TM6SF2 variants—which amplify cardiovascular vulnerability even in the absence of overt obesity. Emerging evidence also links MASLD with an elevated risk of new‐onset heart failure, driven by myocardial steatosis, impaired diastolic relaxation, and chronic low‐grade inflammation that promote the transition to heart failure with preserved ejection fraction (HFpEF) [64, 65]. Taken together, MASLD functions not merely as a hepatic condition but as a multisystem disease with disproportionate cardiovascular impact in Asian populations.

### 2.4 Issues in Specific Populations in East and Southeast Asia

Of important considerations in MASLD are specific populations not given attention especially in the Asian context. Among these are the pediatric population, geriatric population, rural and underserved groups, ethnic minorities, and people with lean physiques but with concurrent MASLD.

There is a rising prevalence of MASLD in the pediatric population in the region, especially countries burdened with high prevalence of obesity [66]. NAFLD in children is highest in Asia in clinical population studies with 62.3% prevalence, almost double the prevalence among their North American counterparts. However, prevalence in the general population remains relatively low at 5.9% [67]. Ultrasound‐based studies show that Asian children have higher prevalence of NAFLD than white children [68]. This rapid increase can be attributed to a multitude of factors such as sugar‐sweetened beverages and foods of low nutritional value, among others [69], as well as suboptimal breastfeeding and complementary feeding practices [70].

Also of particular interest is the lean NAFLD/MASLD population, defined by a body mass index of < 23 kg/m^2^ among Asians, comprising approximately 13% to 20% of cases [71, 72, 73]. This subgroup is more prevalent in East and Southeast Asian countries potentially due to genetic factors such as the PNPLA3 and TM6SF2 polymorphisms [74], genes that modulate insulin sensitivity and regulate intracellular flow of fatty acids and development of fibrosis [75]. Studies comparing non‐lean and lean NAFLD patients showed higher comorbidities and adverse laboratory values among lean NAFLD patients [76]. Diagnosis may often be delayed due to the absence of obesity, highlighting the need for greater awareness among healthcare providers.

Among the elderly, prevalence of MASLD often overlaps with sarcopenia, especially in countries such as Japan and South Korea where diets may not always provide sufficient protein for older adults. Sarcopenia was shown to be independently associated with increased risk of liver fibrosis among middle‐aged and older Chinese population [77], and could indicate poor prognosis of lean NAFLD among the Japanese elders [78]. Among elderly Chinese, high fat‐to‐muscle ratio and low‐BMI were associated with higher risk of MASLD and MASLD‐associated liver fibrosis [79]. Malaysia was noted to have the highest age‐standardized prevalence rate in 2019 with 48,314.12 per 100,000 in Southeast Asia [80]. More than these, the elderly population are predisposed to social isolation, particularly in rural areas of Southeast Asia which can potentially exacerbate sedentary behavior and poor nutrition, further increasing risk for MASLD.

The diversity in the region is heightened by ethnic minorities, especially in Southeast Asians countries such as the Philippines and Malaysia, often having distinct genetic, environmental and lifestyle factors. Traditional diets of root crops or fermented foods as well as overall food disparities may interact uniquely with modern metabolic stressors [81]. Data on this population, however, remains limited in literature.

## 3 Discussion

The adoption of the new nomenclature in 2023, shifting NAFLD to MASLD, introduces a critical challenge as most epidemiological data in East and Southeast Asian countries were collected under the older nomenclature. Thus, existing prevalence, risk factors, and outcome data are not entirely aligned with the refined diagnostic criteria for MASLD. While NAFLD and MASLD essentially refer to the same condition, MASLD acknowledges the connection to metabolic risk factors rather than simply the absence of alcohol consumption. The adoption of the new nomenclature in 2023, shifting NAFLD to MASLD, introduces an important interpretive challenge because much of the epidemiological evidence in East and Southeast Asia was generated under earlier NAFLD‐based definitions. Accordingly, direct comparisons across studies should be made with caution, as case definitions and eligibility criteria may differ between NAFLD‐ and MASLD‐based frameworks.

The diversity in the region compiles a diversity of gaps and challenges, often unique to countries rather than common to the region. Socioeconomic disparities contribute significantly to the burden of MASLD. For instance, urban areas in China, Malaysia and the Philippines are experiencing a surge in obesity‐related MASLD due to sedentary lifestyles and diets rich in fast food and sugary beverages. Meanwhile, rural regions in Vietnam, Indonesia and Myanmar are prone to lean MASLD driven by undernutrition and high‐carbohydrate, low‐protein intake. In‐country differences also exist, further complicating the disparities. East and Southeast Asia are also composed of countries with different income brackets. Japan and Singapore being economically advanced than others, possibly equates to better potential for MASLD management.

We have also seen that pediatric and geriatric populations have limited data on MASLD, although we acknowledge that these groups are gaining wider research efforts in the region. Given the rise of childhood obesity rates and the inclusion of specific criteria in the 2023 nomenclature for MASLD in the pediatric population, early onset MASLD needs to be under consideration and perhaps be better navigated. Moreover, we see limited research on MASLD among women. Recognizing women's health as a key indicator of a country's overall health and well‐being entails thorough understanding of the life events and risk factors unique to women, including hormonal changes, gestation and pregnancy, and overall physical and mental health. Female‐specific risk factors, such as polycystic ovary syndrome (PCOS), pregnancy‐related metabolic changes, and menopause, are under‐researched in Asian populations. Further, the role of estrogen in MASLD pathophysiology and whether postmenopausal women face higher risks require more studies.

The vast archipelagic geography and diverse population of Indonesia and Philippines may prove challenging to the management of MASLD. While remote areas may be protective of the ills of urbanization, it may also hinder early detection and treatment while also resisting interventions. Meanwhile, large countries such as China and Japan may also have regional disparities, urgently calling for a more area‐specific in addition to country‐level epidemiological data. Further, reliance on traditional medicine, while providing accessible care, may further delay proper MASLD management. In countries like Myanmar, Indonesia and rural China [82, 83, 84], traditional healers are often the first point of contact, underscoring the need to engage these practitioners in public health initiatives.

Concretizing this are geographically isolated and disadvantaged areas (GIDA) where limited availability of trained healthcare workers and infrastructures are in place. This gap is further compounded by the logistical difficulties of transporting essential medicines, maintaining equipment, and establishing reliable communication across health networks. More importantly, diagnosis of MASLD should not rely on specialty‐trained physicians.

Especially in the primary healthcare setting, MASLD diagnosis should form part of the competency of primary care physicians as the first point of contact of healthcare systems. Critical to this are non‐invasive diagnostic tools such as scoring systems or ultrasound techniques. For example, the FIB‐4 index, a scoring system that estimates liver scarring, can be used for risk stratification to better inform prognosis and management. However, FIB‐4 still requires blood tests. While simplistic for some countries, this may not be the case for low‐income countries especially in GIDAs. The feasibility of fair and equal access and the capacity of local governments to support remote areas remains constrained by country‐specific socioeconomic and political milieu.

Strengthening regional and national health policies is essential to creating an environment that supports long‐term management of MASLD. Clearer guidelines, backed up with research data, are crucial in understanding the unique epidemiology of MASLD in the region, leading to more tailored interventions. Integration of MASLD into existing health programs for diabetes, hypertension and cardiovascular diseases can streamline delivery of care. In Europe, practice recommendations for the management of MASLD in the primary care setting [85] and joint clinical practice guidelines of specialty societies [86] are available. While these can serve as templates, these may not be totally applicable in the setting of Asia. In most countries in East and Southeast Asia, public health policies and guidelines are often fragmented such that diabetes, hypertension, and other diseases are treated individually. MASLD, however, is an interaction of these and requires recognition as a conglomerate of diseases. A similar guideline on MAFLD by the Asian Pacific Association for the Study of Liver was crafted in 2020, but requires update given the new nomenclature [87]. Specialty societies in Taiwan have collaboratively crafted the first guidance document for patients with diabetes and MASLD, providing practical recommendations for patient care [88].

Lack of research data on the status of specific subpopulations can hinder tailored management of MASLD among Asians. Policies, often patterned from Western counterparts, may not be realistically anchored on cultural and religious sensitivities of the region. Indigenous populations are often excluded from research, leaving their unique needs unaddressed. Needless to say, the government's role, supported by collaborations with NGOs and the private sector, is crucial. Integration of MASLD management into primary healthcare requires capacity building of primary healthcare providers, a surveillance and monitoring system, and an engaging health education and promotion program.

More than productivity losses due to MASLD, the strain on public healthcare systems due to complications could be substantial if MASLD remains unchecked [89]. Ultimately, addressing socioeconomic disparities can substantially can improve health outcomes, not just of MASLD. Adverse social determinants of health in the region can limit access to healthcare, increase food silos, and reduce physical activities, among others. Reducing these inequalities and expanding access to health services can contribute to the broader goal of reducing metabolic diseases in the region and improving the overall quality of life.

## 4 Conclusion and Future Directions

In this region, MASLD presents a growing yet underrecognized public health challenge. Limited awareness, fragmented screening practices, and resource constraints hinder early diagnosis and management. Given the high prevalence of type 2 diabetes, obesity, and other cardiometabolic conditions across East and Southeast Asian populations [90, 91, 92], integrating MASLD into existing non‐communicable disease frameworks is both timely and essential.

To move forward, East and Southeast Asia must prioritize context‐specific strategies: building diagnostic capacity, promoting research tailored to local populations, and fostering regional collaboration among clinicians, researchers and policymakers. Strengthening public health systems and increasing MASLD literacy among healthcare providers will be crucial for improving patient outcomes. In reorienting the clinical and research agenda toward MASLD, East and Southeast Asia could lead in developing inclusive, metabolic‐centered liver care – ensuring that no population is left behind in this evolving landscape of hepatometabolic health.

## Author Contributions

**Jonathan Jaime G. Guerrero:** conceptualization, methodology, data curation, formal analysis, investigation, and writing – review and editing. **Paolo C. Encarnacion:** conceptualization, formal analysis, investigation, and writing – review and editing. **Wan‐Yu Wang:** writing – original draft and writing – review and editing. **Shu‐Fen Liao:** conceptualization and writing – review and editing. **Ching‐Wen Chang:** conceptualization, methodology, data curation, formal analysis, investigation, writing – review and editing, and supervision.

## Ethics Statement

This study was granted approval by the Taipei Medical University ‐ Joint Institutional Review Board (approval number: TMU‐JIRB No. N2023‐10044). All research ethical principles were followed by the authors.

## Conflicts of Interest

The authors declare no conflicts of interest.

## Transparency Statement

The lead author Ching‐Wen Chang affirms that this manuscript is an honest, accurate, and transparent account of the study being reported; that no important aspects of the study have been omitted; and that any discrepancies from the study as planned (and, if relevant, registered) have been explained.

## Supporting information

Supporting File

## Data Availability

The authors have nothing to report.
