# Supplementary material for: Burden and Health System Challenges in MASLD Across East and Southeast Asia: A Narrative Review
Source: Health Sci Rep. 2026 Apr 15;9(4):e72304. doi: 10.1002/hsr2.72304 (PMC13083599; doi:10.1002/hsr2.72304)
Supplement: Supplementary file 1 — Supporting File [file HSR2-9-e72304-s001.docx]

**Supplementary Table 1. Country-level estimates of MASLD, NAFLD, diabetes, obesity, and hypertension across East and Southeast Asia**

| **Country** | **MASLD estimate (as reported in source)** | **NAFLD (2019; age-standardized rate per 100,000)** | **Diabetes (age-standardized rate per 100,000)** | **Obesity (prevalence %, adults ≥18 years)** | **Hypertension (prevalence %, adults 30–79 years)** |
| --- | --- | --- | --- | --- | --- |
| **East Asia** | | | | | |
| China | 36.8%^87^ | 15705.17 | 6157.7 | 6.2% | 27% |
| Japan | 26%^26^ | 7457.49 | 5897.9 | 4.3% | 31% |
| South Korea | 34.23 per 1000^88^ | 8016.73 | 10,346.1 | 4.7% | 27% |
| North Korea | 13,489.7 | 13489.69 | 5430.6 | 6.8% | 27% |
| Taiwan | 23,004.41 | 16553.78 | 5853.6 | 8.2% | 25%^88^ |
| Mongolia | 12,538.35 | 12538.35 | 3975.8 | 20.6% | 43% |
| **Southeast Asia** | | | | | |
| Indonesia | 21707.74 | 21062.21 | 4584.4 | 6.9% | 40% |
| Vietnam | 14,761.69 | 14761.69 | 4518.3 | 2.1% | 30% |
| Laos | 13,511.53 | 13511.53 | 6137.7 | 5.3% | 29% |
| Brunei | 8641.36 | 8303.93 | 13904.0 | 14.1% | 46% |
| Thailand | 24,223.92 | 18376.46 | 5447.8 | 10.0% | 29% |
| Myanmar | 18,130.98 | 18130.98 | 7994.7 | 5.8% | 38% |
| Philippines | 14,396.69 | 14396.83 | 4423.8 | 6.4% | 34% |
| Cambodia | 17022.86 | 17022.86 | 5070.2 | 3.9% | 26% |
| Singapore | 11,966.63 | 9081.92 | 8225.0 | 6.1% | 32% |
| Malaysia | 22,579.65 | 22192.41 | 7416.0 | 15.6% | 41% |
| Timor-Leste | 15,443.92 | 15443.92 | 5902.3 | 3.8% | 35% |

**Note:** Values are presented as reported in the original sources. MASLD estimates were not uniformly available across countries and may be reported either as prevalence (%) or as age-standardized rates, depending on the source; therefore, cross-country comparisons of the MASLD column should be interpreted cautiously. NAFLD and diabetes values are shown as age-standardized rates per 100,000 population. Obesity values represent the prevalence of obesity (body mass index ≥30 kg/m²) among adults aged 18 years or older. Hypertension values represent the prevalence among adults aged 30–79 years. Differences in disease nomenclature, case definition, study year, data source, and estimation method may affect direct comparability across countries. Abbreviations: MASLD, metabolic dysfunction-associated steatotic liver disease; NAFLD, non-alcoholic fatty liver disease; BMI, body mass index.

*Source note: Superscript reference numbers correspond to the citations listed in the supplementary reference section.*
